# AMD3100 inhibits the migration and differentiation of neural stem cells after spinal cord injury

**DOI:** 10.1038/s41598-017-00141-8

**Published:** 2017-03-06

**Authors:** Jia-Ming Liu, Kai Zhao, Liu-Xue Du, Yang Zhou, Xin-Hua Long, Xuan-Yin Chen, Zhi-Li Liu

**Affiliations:** 1 Department of Orthopaedic Surgery, the First Affiliated Hospital of Nanchang University, Nanchang, 330006 PR China; 2 Department of Orthopaedic Surgery, the First Affiliated Hospital of Gannan Medical University, Ganzhou, 341000 PR China

## Abstract

It was reported that CXCR4 signaling played an important role in the migration and differentiation of endogenous neural stem cells after spinal cord injury (SCI). However, the molecular mechanism of it is still unclear. Here, we established a model of SCI in rats and AMD3100 was used to treat them. The rats were then sacrificed and the injured spinal cord specimens were harvested. Additionally, the neural stem cells (NSCs) line was culture and treated with AMD3100 *in vitro*. Results showed the locomotor function of SCI rats was worse after treated with AMD3100. And the expression levels of Nestion in neural stem cells and β-tubulin in neuron cells were significantly increased in the injured spinal cord, which can be inhibited by the CXCR4 antagonist of AMD3100. Additionally, the expression of β-catenin and phosphorylase β-catenin protein was significantly down regulated by AMD3100. *In vitro*, the NSCs proliferation ability was inhibited and the migration was decreased after treated with AMD3100. Also, the expression of Nestion, β-tubulin, β-catenin and phosphorylase β-catenin protein was significantly decreased in AMD3100 group comparing with untreated group. Taken together, this study suggested that AMD3100 could inhibit the migration and differentiation of endogenous neural stem cells in rats with SCI. The mechanism of it maybe that AMD3100 could down regulate of SDF-1/CXCR4 by targeting β-catenin signaling pathway.

## Introduction

Spinal cord injury (SCI), which is a leading cause of disability in modern society, commonly results from trauma. The pathophysiology of the SCI is a complex issue which will affect the nervous, vascular and immune systems^1^. SCI can cause patients’ sensory, motor and autonomic nerve dysfunction and reduce the quality of patients’ life. The treatment for SCI, including surgical decompression and methylprednisolone treatment, can only block the secondary SCI occurrence, and the neurologic function of patients couldn’t be restored due to injured spinal cord cells^2^. Although studies have found that the proliferation, migration and differentiation of endogenous neural stem cells were detected in the injured spinal cord, the process of it is limited and only a small number of stem cells can differentiate into neuron cells with neurologic function^3^. Therefore, how to promote the regeneration of nerve cells after SCI is important for patients.

CXCR4 is a α-chemokine receptor of stromal-derived-factor-1 (SDF-1), which plays an important role in lymphocyte chemotaxis^4^. The activation of CXCR4 by its chemokine ligand CXCL12 regulates a variety of physiopathological functions in the central nervous system. Current studies suggested that CXCR4 signaling also played an important role in the migration and differentiation of endogenous neural stem cells^5^. Although the intracellular expression level of CXCR4 was very low in normal tissues, it significantly increased in injured spinal cord^6^. However, the role and molecular mechanism of CXCR4 for the migration and differentiation of endogenous neural stem cells after SCI is still unclear.

AMD3100 is a specific antagonist to the CXCR4 receptor^7^. It was initially used for inhibition HIV virus entry^8^, but now it was used as a hematopoietic stem cell mobilization agent^9^. Here, in this study, we hypothesized that inhibition of CXCR4 by AMD3100 would inhibit the migration and differentiation of neural stem cells via down-regulating β-catenin signaling pathway.

## Results

### The SDF-1/CXCR4 axis was evoked after spinal cord injury in rats

**Figure 1:**
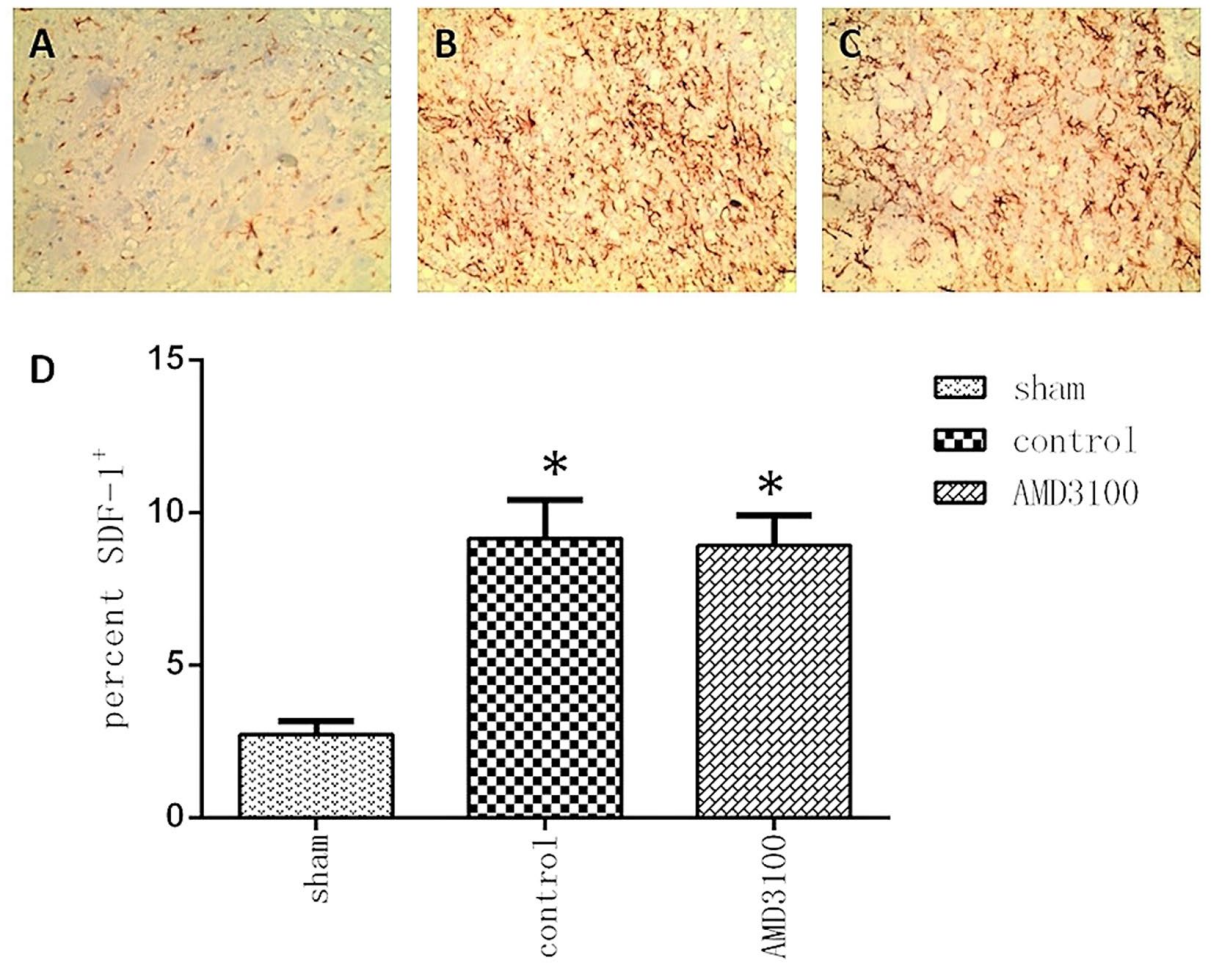
The protein expression of SDF-1 up-regulated in neural stem cells of the lesion site on the 7th day after surgery. (**A**) Representative image of IHC staining of SDF-1 in sham group (×400). (**B** and **C**) Representative images of IHC staining of SDF-1 in untreated and AMD3100 groups (×400). The expression of SDF-1 protein increased in the neural stem cells after SCI. (**D**) The expression of SDF-1 was significant higher in untreated and AMD3100 groups than those in sham group. (*P < 0.05: untreated group vs. sham group, AMD3100 group vs. sham group).

In order to investigate whether SCI could evoke the activity of SDF-1/CXCR4 axis in rat, the expression of SDF-1 protein was detected by immunohistochemstry (IHC) assay. The results showed SDF-1 protein was expressed in nuclei of neural stem cell and was significantly higher in SCI group than those in sham group (Fig. 1). It suggested that SCI could evoke the activity of SDF-1/CXCR4 axis.

### Inhibition of SDF-1/CXCR4 could suppress the recovery of neurologic function in rats with SCI

**Figure 2:**
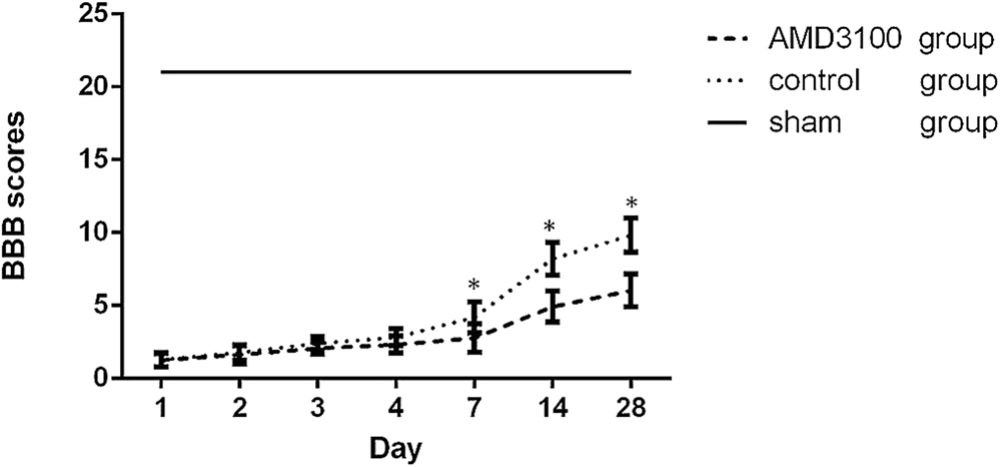
The BBB scores of the rats during the 28 days period after surgery in the three groups. It showed that the scores were significantly lower in AMD3100 group than those in untreated group on the 7th, 14th and 28th day after SCI. (*P < 0.05).

For determining the effect of inhibiting SDF-1/CXCR4 on the recovery of neurologic function after spinal cord injury, we used AMD3100 to treat the rats with SCI. And the neurologic function of rats was measured by the Basso-Beanie-Bresnahan (BBB) score at different time points. It showed that the BBB scores in both of untreated and AMD3100 groups improved as time going on. However, they were significantly lower in AMD3100 group than those in untreated group at different time points (Fig. 2). It indicated that inhibition of SDF-1/CXCR4 could suppress the recovery of neurologic function in rats with SCI.

### Inhibition of SDF-1/CXCR4 could suppress the proliferation of neural stem cells *in vitro*

**Figure 3:**
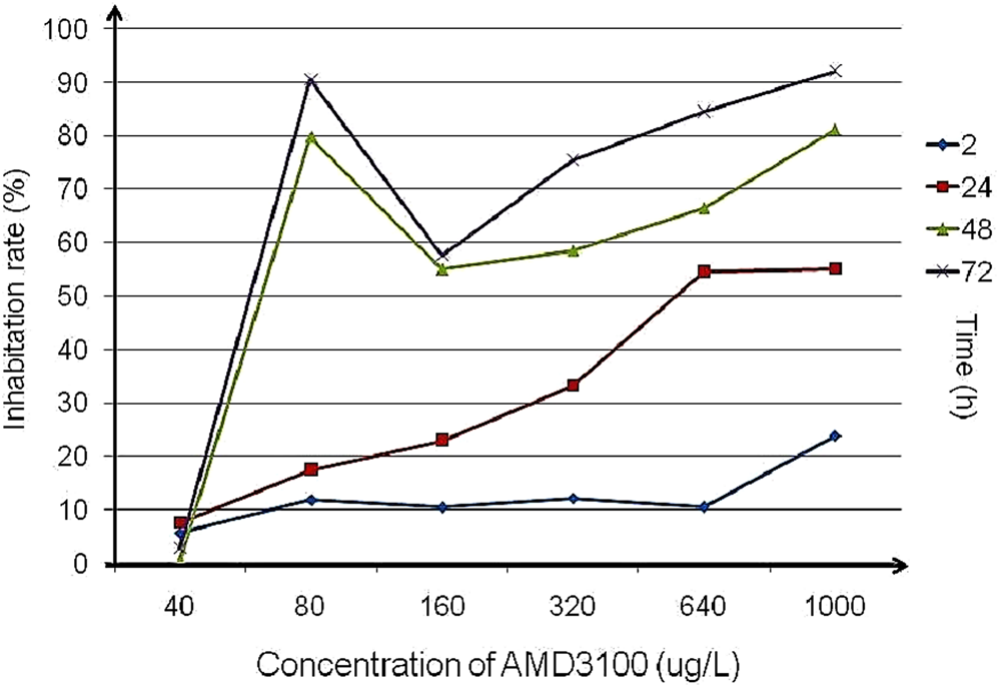
The proliferation of neural stem cells treated by AMD3100 *in vitro*. As the time going on, the inhabitation rates of neural stem cells proliferation were gradually increased.

The proliferation of neural stem cells (NSCs) inhibited by AMD3100 was evaluated by CCK-8 assay *in vitro*. The NSCs line of RASNF-01001 cells (Cyagen, USA) was treated with different concentrations of AMD3100. As shown by the CCK-8 assay, the CXCR4 antagonist of AMD3100 significantly decreased the proliferation ability of NSCs as the time going on (Fig. 3). The results showed that inhibition of SDF-1/CXCR4 could suppress the proliferation of NSCs *in vitro*.

### Inhibition of SDF-1/CXCR4 could suppress the migration of NSCs *in vivo* and *vitro*

**Figure 4:**
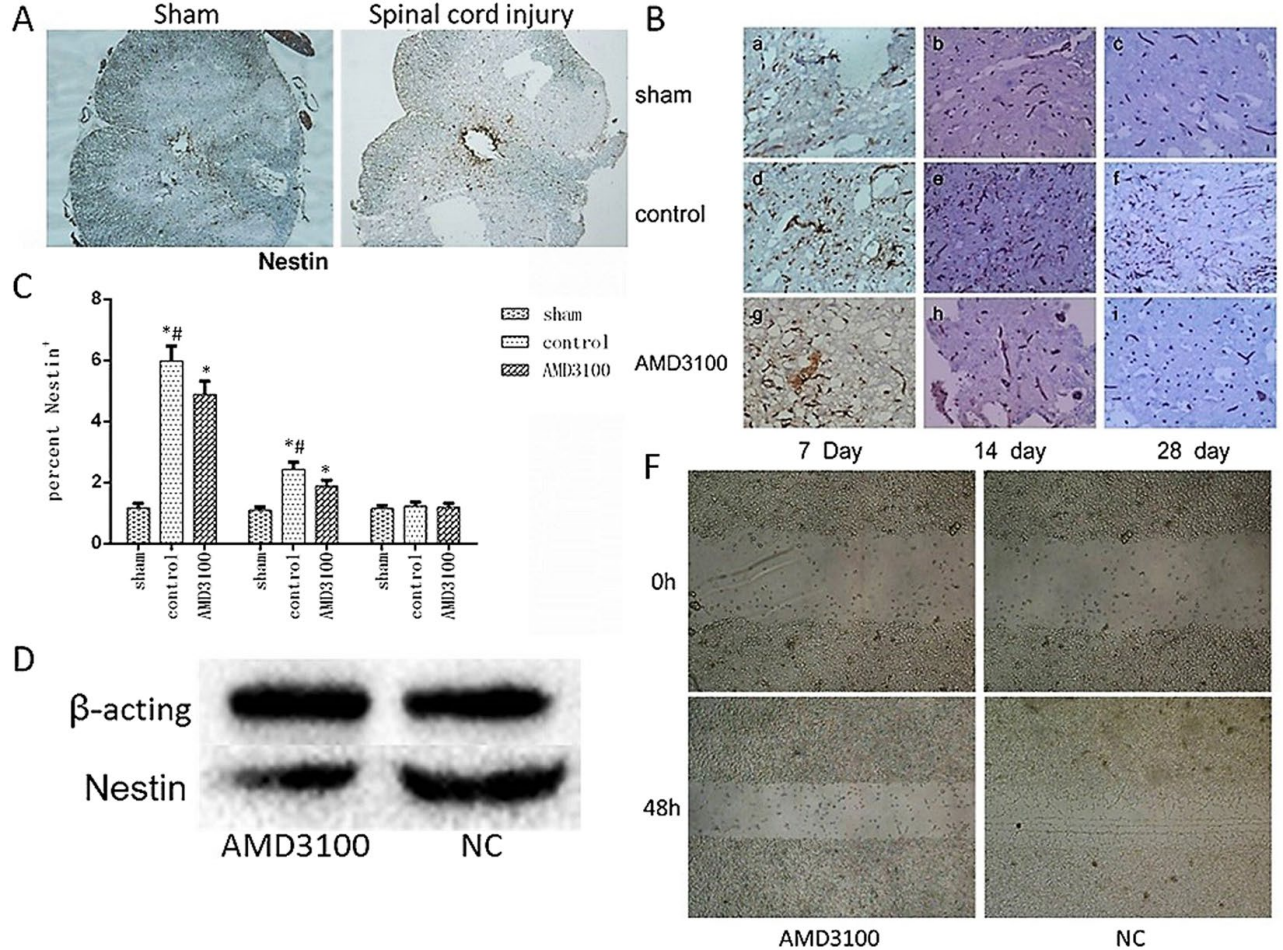
The expression of Nestin protein in neural stem cell after treated with AMD3100. (**A**) Representative images of IHC staining of Nestin in sham and untreated groups of rats (×40). It showed that the expression of Nestin protein increased at the injured site after SCI in rats comparing with those without SCI, especially in the region around central canal of spinal cord. (**B**) Representative images of IHC staining of Nestin in the three groups of rats (×400). It showed that the expression of Nestin increased in both of AMD3100 and untreated groups after SCI. (**C**) The expression of Nestin protein was measured in the three groups of rats. It revealed that the Nestin protein was inhibited by AMD3100. (**D**) The expression of Nestin protein in RASNF-01001 cells was significantly decreased in AMD3100 group. (**F**) The wound healing assay showed the migration ability of RASNF-01001 cells was reduced by AMD3100. (*P < 0.05: untreated group vs. sham group, AMD3100 group vs. sham group; ^#^ *P* < 0.05: AMD3100 group vs. untreated group).

First of all, in order to detect the effect of inhibiting SDF-1/CXCR4 on the migration of endogenous neural stem cells in rats with SCI, the neural stem cells in the region of injured spinal cord were counted on the images of IHC in terms to the expression of Nestin protein. Nestin is a special biomarker of neural stem cell^10^ and its protein was expressed in the cyton of neural stem cell **(**Fig. 4A–C **)**. The results showed that the expression of Nestin protein significantly increased on the 7th day after surgery and gradually decreased from the 7th to the 28th day in both of untreated and AMD3100 groups. However, the expression of Nestin was significantly lower in AMD3100 group comparing with that in untreated group.

Secondly, the RASNF-01001 cells of NSCs were treated with AMD3100 *in vitro*. And the protein expression of Nestin was measured by western blotting assay. The resulted displayed that the expression of Nestin protein in AMD3100 group was significantly decreased comparing with that in control group (Fig. 4D, *P* < 0.05).

Finally, in order to determine the influence of AMD3100 on the migration of NSCs *in vitro*, we used wound healing assay to evaluate the migration ability of RASNF-01001 cells after treated with AMD310. We found that inhibition of CXCR4 by AMD3100 significantly reduced the ability of RASNF-01001 cells migration comparing with untreated group (Fig. 4F). All the results revealed that inhibition of SDF-1/CXCR4 could suppress the migration of NSCs *in vivo* and *vitro*.

### Inhibition of SDF-1/CXCR4 could suppress the differentiation of NSCs *in vivo* and *vitro*

**Figure 5:**
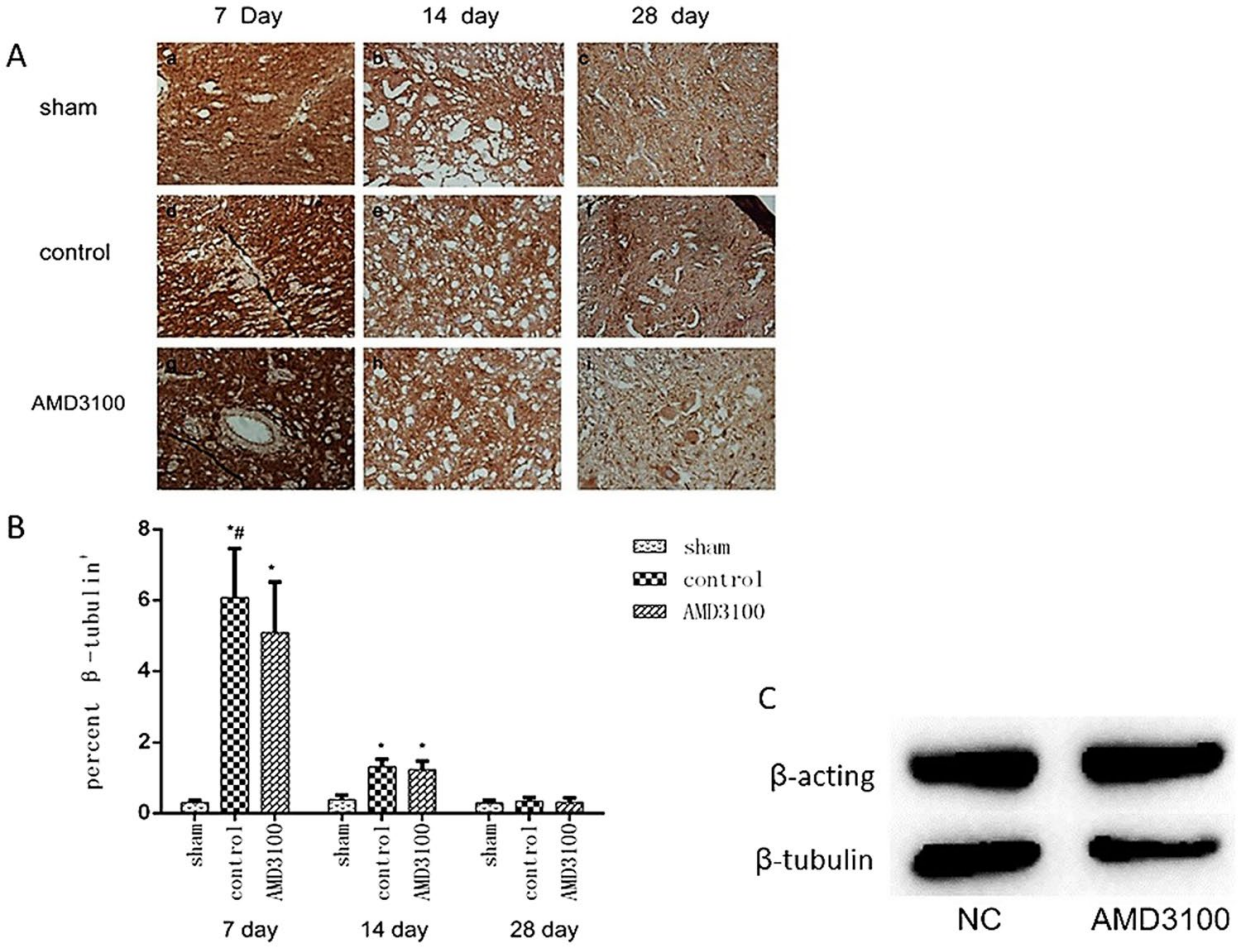
The expression of β-tubulin protein in neural stem cell after treated with AMD3100. (**A**) Representative images of IHC staining of β-tubulin in the three groups of rats (×400). It showed that the expression of β-tubulin increased on the 7th day and then decreased from the 7th to 28th day after surgery. (**B**) The expression of β-tubulin protein was lower in AMD3100 group of rats than that in untreated group after surgery. (**C**) The expression of β-tubulin protein in RASNF-01001 cells was significantly lower than that of control group after treated with AMD3100. (*P < 0.05: untreated group vs. sham group, AMD3100 group vs. sham group; ^#^ *P* < 0.05: AMD3100 group vs. untreated group).

For the purpose of identifying the effect of inhibition of SDF-1/CXCR4 on the differentiation of endogenous neural stem cells in rats with SCI, the number of neurons cells in the region of injured spinal cord were measured on IHC images according to the expression of β-tubulin protein, which was a specially biomarker of neurons cells. The results showed that β-tubulin protein was expressed in cyton of neurons cells (Fig. 5A,B). And it significantly increased on the 7th day after surgery, and it then decreased. Additionally, the expression of β-tubulin was higher in untreated group than that in AMD3100 group at different time points after surgery.

Additionally, we used AMD3100 to treat RASNF-01001 cells *in vitro*, and the expression of β-tubulin protein was measured by western blotting assay. The results demonstrated that the protein expression level of β-tubulin in AMD3100 group was significantly lower than that of control group (Fig. 5C). These results indicated that inhibition of SDF-1/CXCR4 by AMD3100 could suppress the differentiation of NSCs *in vivo* and *vitro*.

### Inhibition of CXCR4 could down-regulate the activity of β-catenin *in vivo* and *vitro*

**Figure 6:**
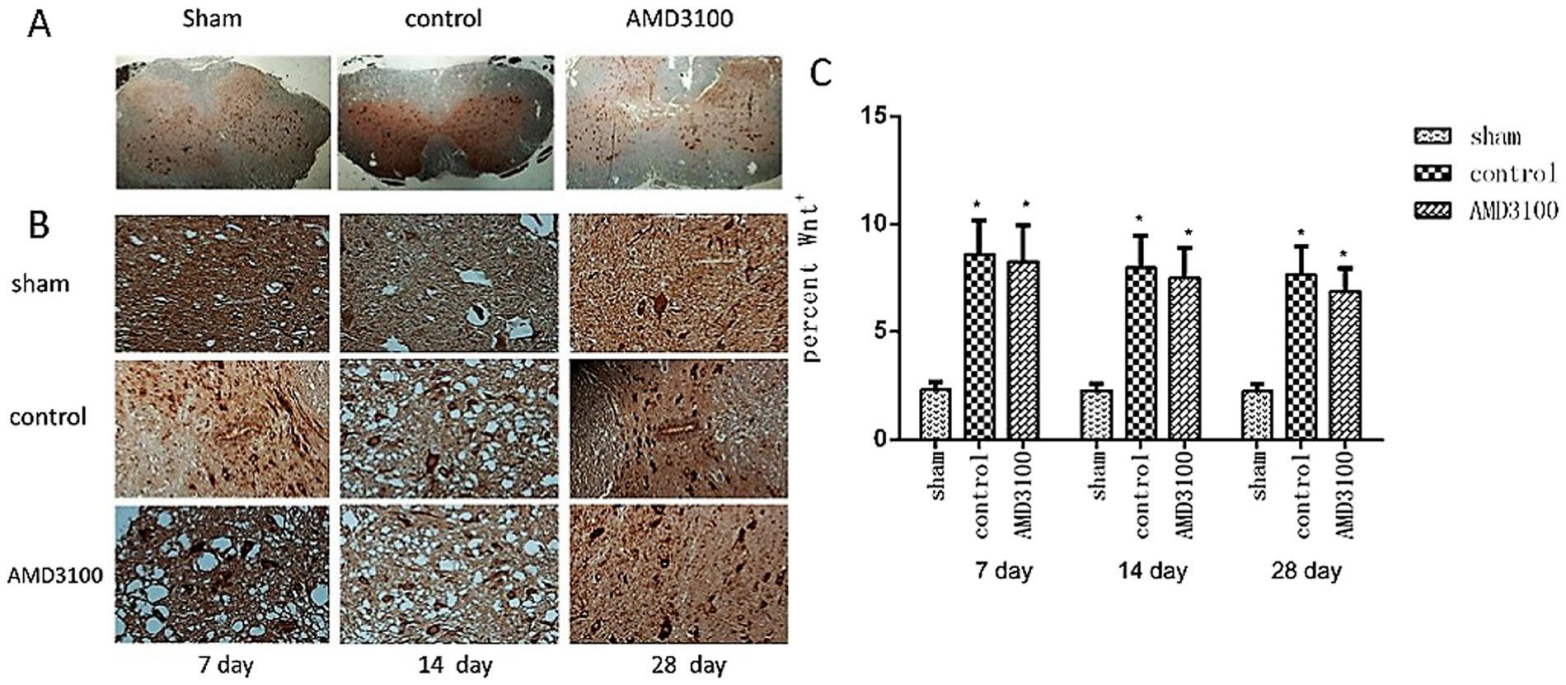
The expression of Wnt protein in the three groups of rats after surgery. (**A**) Representative images of IHC staining of Wnt in the three groups on the 7th day after surgery (×40). (**B**) Representative images of IHC staining of Wnt in the three groups (×400). It showed that the expression of Wnt protein in AMD3100 and untreated groups increased after surgery. (**C**) The expression level of Wnt protein was significantly higher in AMD3100 and untreated groups than those in sham group on the 7th, 14th and 28th day after surgery. However, no different was found for Wnt between AMD3100 and untreated groups at the different time. (*P < 0.05: untreated group vs. sham group, AMD3100 group vs. sham group).

**Figure 7:**
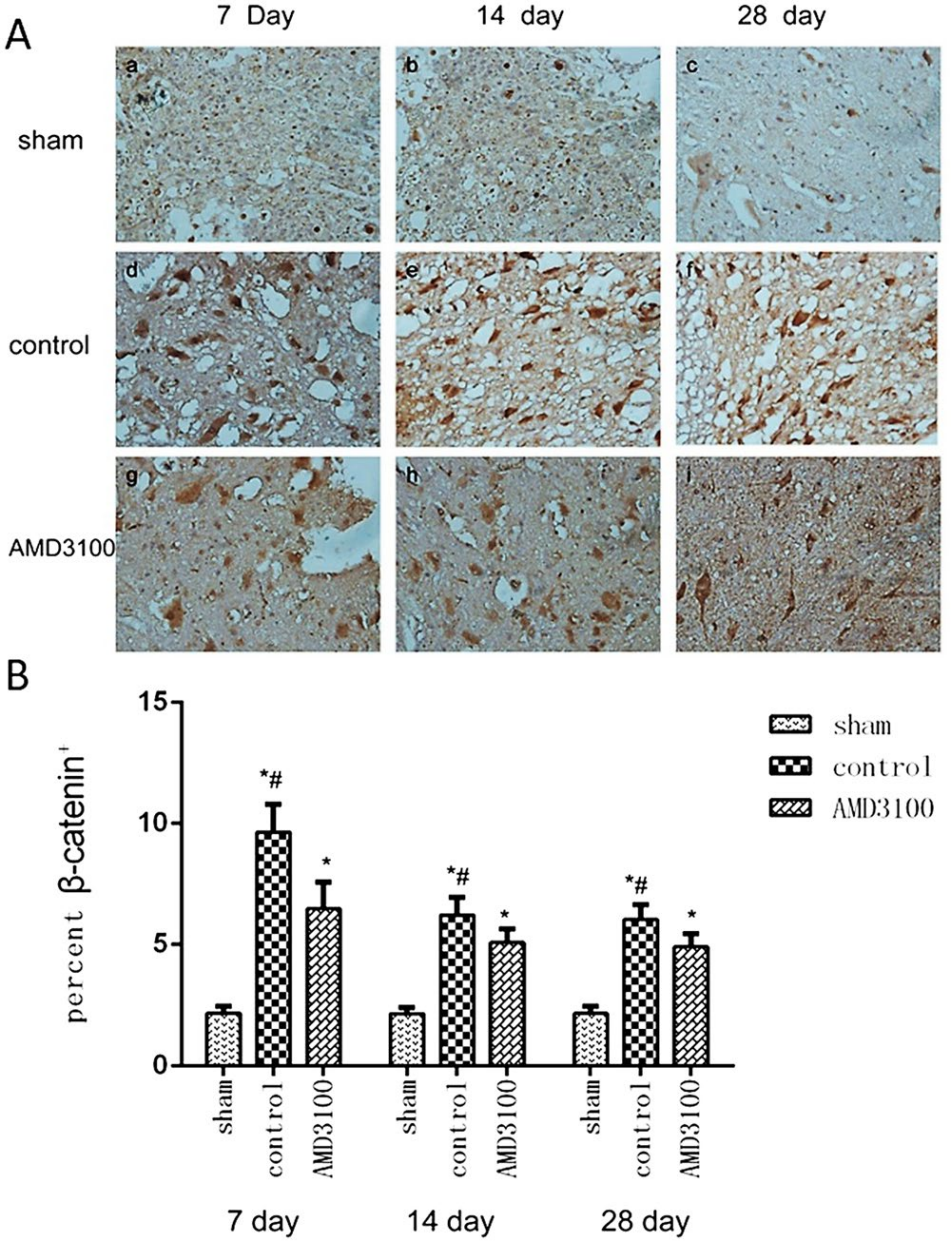
The expression of β-catenin in the three groups of rats after surgery. (**A**) Representative images of IHC staining of β-catenin in the three groups (×400). It showed that the expression of β-catenin increased in both of AMD3100 and untreated groups. (**B**) The expression of β-catenin protein was measured and the expression of it was significantly lower in AMD3100 group comparing with that in untreated group at the different time after surgery. (*P < 0.05: untreated group vs. sham group, AMD3100 group vs. sham group; ^#^ *P* < 0.05: AMD3100 group vs. untreated group).

**Figure 8:**
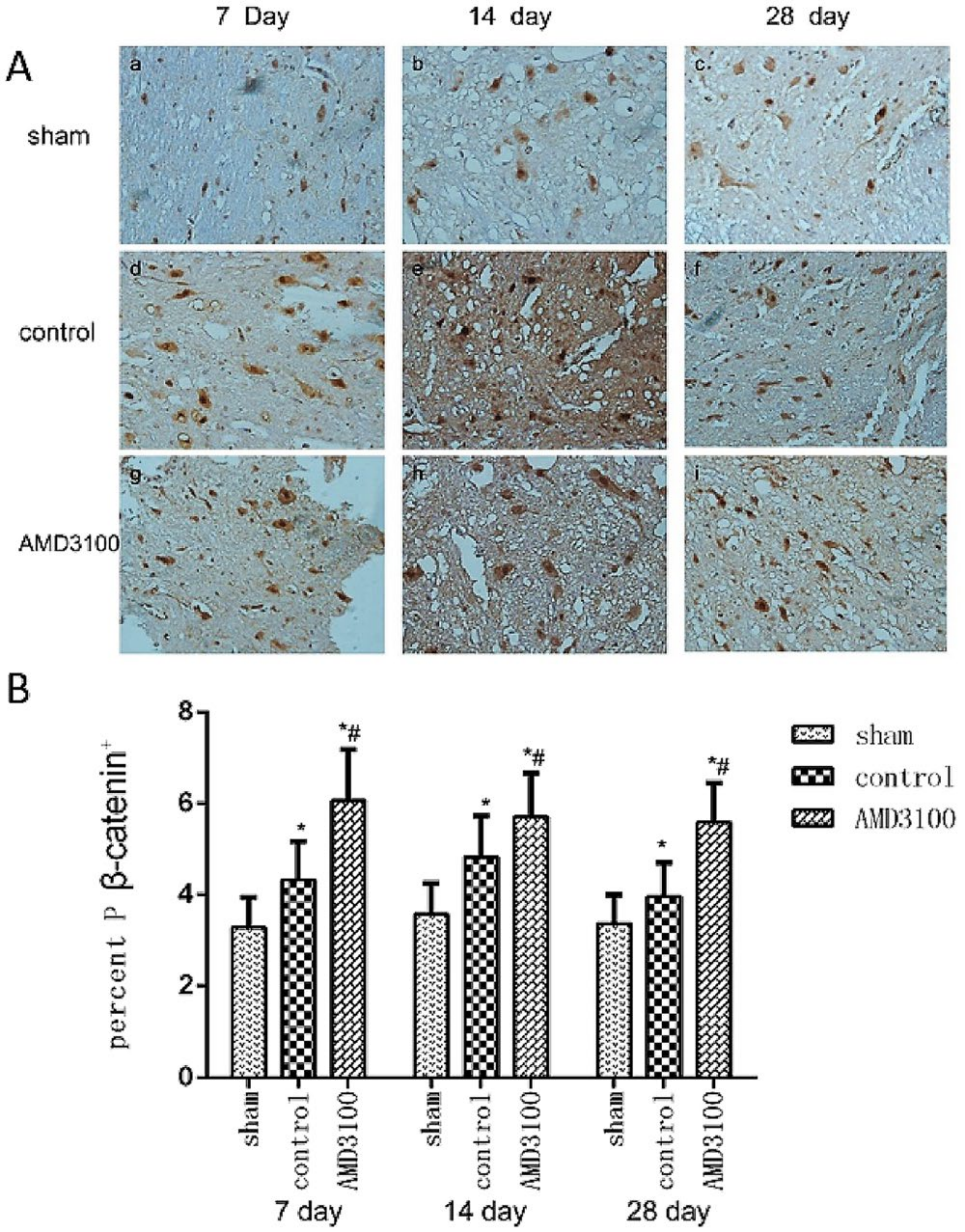
The expression of phosphorylase β-catenin protein in the three groups of rats after surgery. (**A**) Representative images of IHC staining of phosphorylase β-catenin in the three groups (×400). It showed that the expression of phosphorylase β-catenin also increased in both of AMD3100 and untreated groups. (**B**) The expression level of phosphorylase β-catenin was significantly higher in AMD3100 group than that in untreated group on the 7th, 14th and 28th day after surgery. (*P < 0.05: untreated group vs. sham group, AMD3100 group vs. sham group; ^#^ *P* < 0.05: AMD3100 group vs. untreated group).

For investigating the effect of inhibition of SDF-1/CXCR4 on the activity of Wnt/β-catenin signaling pathway, the expression of Wnt and β-catenin protein in injured spinal cord was measured by IHC assay. Wnt and β-catenin proteins were expressed in the cyton of neural stem cells. The expression of Wnt in neural stem cells significantly increased in both of AMD3100 and untreated groups after the surgery. However, no significant difference was noted for Wnt protein between the two groups (*P* = 0.87) (Fig. 6). The expression level of β-catenin was significant lower in AMD3100 group than that in untreated group (Fig. 7). Moreover, we also used the phosphorylase of β-catenin to confirm our results (Fig. 8).

**Figure 9:**
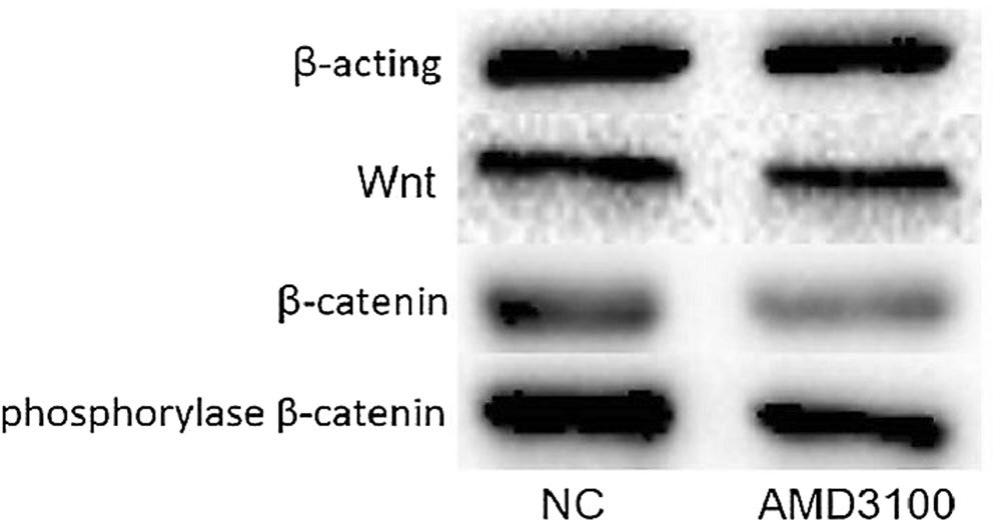
The protein expression of Wnt, β-catenin and the phosphorylase of β-catenin in RASNF-01001 cells after treated with AMD3100. The expression levels of β-catenin and the phosphorylase of β-catenin were decreased in AMD3100 group, but the expression Wnt was not significantly changed after treated with AMD3100.

Moreover, in order to detect the effect of CXCR4 on Wnt/β-catenin signaling pathway in NSCs *in vitro*, western blotting assay was used to access the protein expression levels of Wnt, β-catenin and the phosphorylase of β-catenin in neural stem cell line of RASNF-01001 after treat with AMD3100. The results showed that the expression of Wnt protein in AMD3100 group was not significantly different from the control group (*P* > 0.05). However, the proteins of β-catenin and phosphorylase β-catenin expressed in AMD3100 group were significantly lower than those in control group (*P* < 0.05) (Fig. 9). These outcomes indicated that inhibition of CXCR4 could down regulate β-catenin signaling pathway in NSCs.

## Discussion

Chemokine plays an important role in regulating cell migration. SDF-1 is a kind of chemotactic factor for many cell types and CXCR4 is the receptor of it, which expresses on the surface of different kinds of stem cells^11^. It has been reported that SDF-1/CXCR4 axis constitutively expresses in a lot of tissues, such as the brain, kidney, heart, liver and spleen^12^. And it is also involved in several malignant tumors, such as breast, colon and pancreatic cancers^13–15^. Moreover, several studies showed that the interaction of SDF-1 with its receptor CXCR4 played a part in mediating mesenchymal stem cell (MSC) migration to the injured site^16, 17^. Li *et al*.^18^ showed that the SDF-1/CXCR4 axis maintained neural stem cells and worked as a salvage signal pathway for initiating endogenous stem cell-based tissue repair. Tysseling *et al*.^19^ demonstrated that attraction of CXCR4+ macrophages was a part of programmed response to injury and modulation of the SDF-1 signaling system may be important for regulating the inflammatory response after SCI. In line with Tysseling *et al.* study, in the present study, we found that the expression of SDF-1 was significantly higher in injured spinal cord section than those in uninjured spinal cord in rats. It indicated that SDF-1/CXCR4 axis was evoked in injured spinal cord and it may play an important role in spinal cord repair.

In order to investigate the potential effect of SDF-1/CXCR4 expression after SCI, the rats were treated with AMD3100. AMD3100 was proved to drive endogenous stem cells from the bone marrow (BM) to the blood stream in animals and human^20^. Liu *et al*.^21^ indicated that CXCR4-mediated migration of BMSCs can be inhibited by AMD3100 in ischemic kidneys repair. In the present study, we used Basso-Beattie-Bresnahan score to assess the locomotor functions of the rats after SCI. After inhibition of CXCR4 by AMD3100, the locomotor functions of the rats significantly declined. It indicated that inhibition CXCR4 would suppress the injured spinal cord repair, which was similar to the results of Liu *et al.’*s study.

Nestin is a member of type VI intermediate filament protein family, which widely expresses in mammalian nervous tissue and stem cells of non-neuronal normal tissues^22^. It was originally described as a neural stem cell marker during central nervous system development^23^. In the current study, we found there was an increasing deposition of the percentage of Nestin-positive neural stem cells around the central canal of the spinal cord, which indicated the neural stem cells would migrate to the injured site of the cord after SCI. However, after giving AMD3100 to the rats, the expression of Nestin decreased at the injured site of spinal cord. Moreover, the experiment *in vitro* also showed that the protein expression of Nestin was significantly decreased in NSCs after treated with AMD3100. We also found that the migration ability of NSCs was inhibited by AMD3100 *in vitro* experiment. Li *et al*.^24^ showed that SDF-1/CXCR4 axis could promote the migration of mesenchymal stem cells to the lesion site following SCI. Thus, we believed that the migration of endogenous neural stem cells to the injured site of spinal cord after SCI was mediated by CXCR4.

As one of the members of the cytoskeleton family, the distribution of class III β-tubulin is a popular identifier specific for neurons in nervous tissue^25^. In our study, the expression level of β-tubulin was increased after SCI. However, after inhibiting CXCR4 with AMD3100, the expression of β-tubulin protein significantly decreased during the 28 days period after SCI. And the results were confirmed *in vitro* experiment. It suggested that CXCR4 was able to mediate the differentiation of endogenous neural stem cells to neuron cells, which could be inhibited by AMD3100. Based on the results, it’s helpful for injured spinal cord repair if we can promote the activity of SDF-1/CXCR4 axis.

Although the migration and differentiation of endogenous neural stem cells mediated by SDF-1/CXCR4 axis after SCI was detected in this study, the molecular mechanism of CXCR4 regulating neural stem cells after injury is still unclear. Previous studies have found positive regulation between SDF-1/CXCR4 and β-catenin in several malignant tumors^26^. Recently, Luo *et al*.^27^ demonstrated that SDF-1/CXCR4 axis greatly increased the accumulation of β-catenin in neural progenitors and played crucial roles in the development of the central nervous system. In the present study, we found the expression of β-catenin in SCI could be inhibited by the CXCR4 antagonist of AMD3100 *in vivo* and *vitro*, which suggested SDF-1/CXCR4 axis might regulate β-catenin signaling pathway in neural stem cells for the migration and differentiation.

Limitations were also existed in this study. For example, the methodologies used *in vivo* and *vitro* experiments were limited, and the data for the opinion that β-catenin signaling pathway was involved in the mechanism of AMD3100 inhibition of NSCs migration and differentiation were not enough. Additional methodologies and experiments were needed to support the results of our study.

In conclusion, based on this study, the activity of SDF-1/CXCR4 axis could be evoked by SCI, and inhibition the expression of CXCR4 by AMD3100 could suppress the migration and differentiation of neural stem cells. The mechanism for it maybe AMD3100 could down regulate SDF-1/CXCR4 by targeting β-catenin signaling pathway. This is the first study linking chemokine expression with β-catenin signaling pathway in SCI and the outcomes found in this study may be helpful for the treatment of SCI. However, the potential molecular mechanism of SDF-1/CXCR4 regulating the expression of β-catenin in SCI is still not clear, and further study is needed.

## Materials and Methods

### Experimentation on animals

Adult female Sprague-Dawley (SD) rats (weighting 220–250 g) were obtained from the laboratory animal science centre of the Nanchang University (Nanchang, China) for the experiments. All the procedures and protocols were approved by the Ethics Committee on Animal Experiments of the first affiliated hospital of Nanchang university. The rats were housed 4 per cage with free access to food and water and maintained in a suitable environment at 25 °C and a 12 hour light/dark cycle. All animal procedures and maintenance were conducted in accordance with the institutional guidelines of the university. Rats were anesthetized via an intraperitoneal injection of 2% pentobarbital (2 ml/kg). And then the spinous process and vertebral lamina were removed to expose a circular region of dura at the thoracic 10 vertebral level^28^. An incomplete SCI was made by dropping a 10 grams metal rod onto the dura from a height of 25 mm^29^. Rat’s bladders were manually emptied three times a day until the reflex bladder emptying function was restored. Thirty-six rats were randomly assigned into 3 groups (n = 12/group): i) the sham-operated group; ii) the untreated group (intraperitoneal injection of 5 mg/kg PBS for five days); iii) the AMD3100 group (intraperitoneal injection of 5 mg/kg AMD3100 for five days). Rats were sacrificed on the 7th, 14th, 21th and 28th day after surgery, respectively.

### Motor behavioral analysis

The Basso-Beanie-Bresnahan (BBB) score^30^ was used to assess the locomotor function of rats after SCI. The function assessment were carried out by two researchers independently, who blinded to the treatment of each group. The rats were tested on days 1, 2, 3, 4, 7, 14 and 28 after the surgery, respectively. And the BBB scores were recorded.

### Immunohistochemstry assay

All the rats were sacrificed following anesthesia as mentioned above and perfused through the ascending aorta with saline, following by 4% paraformaldehyde. A 1.0 cm section of spinal cord at the injured site was dissected out, dehydrated through a graded alcohol series for 24 hours, and sliced into 20-μm-thick longitudinal frozen sections. Sections were permeabilized by incubation in 0.3% Triton X-100 for 30 minutes in phosphate buffer saline (PBS) at room temperature. The sections were rinsed thrice with PBS and incubated with primary antibody overnight at 4 °C. Antibodies used as follows: rabbit anti-beta catenin (1:2000, abcam, ab6302), mouse anti-nestin (1:500, abcam, ab6142), rabbit anti-wnt1 (1:1000, abcam, ab85060), rabbit anti-beta-tubulin (1:500, origene, TA306985) and rabbit anti-polyconal beta-catenin (1:100, origene, TA313140). Finally, Image-Pro Plus 6.0 (Media Cybernetics, Silver Spring, MD, USA) was used for quantitative analysis on the immunohistochemstry (IHC) images. During image acquisition, the illumination level of each imaging session was maintained by stabilizing the light source. The area ratio of positive protein was used to quantify the expression of the target protein on IHC images.

### Cell culture

The Sprague-Dawley rat neural stem cells (NSCs) line of RASNF-01001 was purchased from the company of Cyage (USA), and the cells were cultured in RASNF-90011 medium (Cyage, USA) at 37 °C in a humidified atmosphere of 5% CO2. After the neural spheres formation and getting large, the passage was carried out.

### Cell proliferation assay

The NSCs proliferation ability was determined with the Cell Counting Kit-8 (CCK-8) assay (TransGen, China). The Cells were cultured in 96-well tissue culture plates at a cell density of 1 × 10^4^ cells/well. The plate was incubated for 24 h at 37 °C in a humidified atmosphere of 5% CO2. After added AMD3100 to the medium, the cells were incubated for 24, 48 and 72 h. Then, 10 µL CCK-8 solution were added into each well and further cultured for 2 h. The optical density (OD) was measured at 490 nm wave length. The experiments were repeated three times over multiple days.

### Western blotting

Western blotting assay was used to detect the protein expression of Nestin, β-Tubulin, Wnt1, β-Catenin and phosphorylase of β-Catenin (Phospho-Tyr489). After treated with AMD3100, the cellular lysates of RASNF-01001 were obtained using RIPA lysis buffer containing 6 μg/ml PMSF. Total protein was extracted and the protein concentration was determined by Bradford assay. Western blotting assay was carried out using antibodies against Nestin (Signalway Antibody, USA), β-tubulin (Signalway Antibody, USA), Wnt1 (Abcam, USA), β-catenin and phosphorylase β-catenin (Phospho-Tyr489) (Signalway Antibody, USA). The immune complexes were measured with pro-light HRP Kit (TIAN GEN, China). Expression of proteins was analyzed using ImageJ software (NIH, Bethesda, MD, USA). All the experiments were repeated three times over multiple days.

### Wound healing assay

The ability of NSCs migration was assessed by wound healing assay. The cells were grown to confluence in 6-well tissue culture plate at a density of 5 × 10^6^ cells/well after treated with AMD3100. The cells were then denuded by dragging a rubber policeman (Fisher Scientific, Hampton, NH, USA) through the center of the plate. And the cells were incubated at 37 °C for 72 h. ImagesJ software was used to measure the distance of the NSCs migration in the well at 24, 48 and 72 h, respectively. All the experiments were repeated three times over multiple days.

### Statistical analysis

All measurement data were presented as $\bar{X}$ ± SD. One-way *ANOVA* and least significant difference (LSD) test were used for statistical analysis. A *P* value of <0.05 was considered to be statistically significant. All analysis was performed using SPSS version 13.0 (Statistical Software for Social Sciences, Inc., Chicago, IL, USA).
